# Cellular Signal Transduction Pathways by Leptin in Colorectal Cancer Tissue: Preliminary Results

**DOI:** 10.5402/2011/575397

**Published:** 2011-03-28

**Authors:** Ewa Nowakowska-Zajdel, Urszula Mazurek, Malgorzata Stachowicz, Elzbieta Niedworok, Edyta Fatyga, Małgorzata Muc-Wierzgoń

**Affiliations:** ^1^Department of Internal Diseases, Medical University of Silesia, Żeromskiego 7 Street, 41-902 Bytom, Poland; ^2^Department of Molecular Biology, Medical University of Silesia, Narcyzów 1 Street, 41-200 Sosnowiec, Poland; ^3^Department of Human Nutrition, Medical University of Silesia, Piekarska 18 Street, 41-902 Bytom, Poland

## Abstract

The aim of the study was to analyse genes typing with the use of the oligonucleotide microarray technique (HG-U133A, Affymetrix) differentiating colorectal cancer tissues from tissues assessed histopathologically as healthy ones among a panel of 91 mRNA of genes encoding proteins involved in activation of cellular signal transduction pathways by leptin. Frozen tumor specimens from 11 colon cancer patients in various stages of clinical progression of the disease in an I–IV stage scale according to the TNM staging were used in molecular tests. Among the genes participating in the cascade of signal transfer in cell activated by leptin, the following ones: AKT1, STAT3, MCL1 were qualified as differentiating stage I and II and VEGFC, CCNDI the encoding genes respectively as differentiating III and IV stage neoplasm. It is necessary to extend studies of analysis of cellular signal transduction pathways by leptin in colorectal cancer initiation and transformation processes.

## 1. Introduction

Leptin is a pleiotropic adipokine whose expression level and the level of release to blood correlate to a large extent with the amount of fat tissue in the organism. Numerous researchers [1–5] stress the role of leptin and its receptor, Ob-R, in induction and progression of various cancer types. Experimental data reveal that leptin may stimulate—through activation of cellular signal transduction pathways—cell growth, inhibit apoptosis processes, and induce migration processes and angiogenic factor expression in numerous cancer types. Leptin significantly enhanced the migratory activity of various human colon carcinoma cell lines such as SW480, SW620, and HCT116. The strongest effect was observed in SW480 cells, which increased their locomotor activity from 28% spontaneously locomoting cells to 50% [6].

 The pleiotropic nature of leptin is related to presence of the leptin receptor, Ob-R, in the organism. Several isoforms of the leptin receptor are known. These result from alternative exon splicing and proteolytic protein splitting in the already existing isoforms [7]. These are Ob-R: Ob-Ra, Ob-Rb, Ob-Rc, Ob-Rd, and OB-Rf and the soluble form of the Ob-Re receptor which is responsible for transport of leptin across blood [8–10]. 

 The aim of the study was to analyse genes typing with the use of the oligonucleotide microarray technique (HG-U133A, Affymetrix) differentiating colorectal cancer tissues from tissues assessed histopathologically as healthy ones among a panel of 91 mRNA of genes encoding proteins involved in activation of cellular signal transduction pathways by leptin.

## 2. Materials and Method

Frozen tumor specimens from 11 (2 coded Stage I, 2 Stage II, 2 Stage III, and 2 Stage IV and 3 coded control group) colon cancer patients were obtained. All the patients were informed about the planned study, and their informed consent in writing was obtained. 

The histopathology of each specimen was reviewed to confirm diagnosis and tumor content. Tumor content was estimated in percentage by counting nuclei of epithelial tumor cells. Patient eligibility criteria include colon primary stages I–IV adenocarcinoma, primary treatment is surgery only without adjuvant or neoadjuvant therapy, at least 70% of tumor cells in the tissue sample.

The Bioethical Commission of the Medical University of Silesia expressed its consent to perform the tests.

### 2.1. Method

In the study, intraoperatively collected large intestine specimens affected by cancer of a size of 1 cm^3^, as well as healthy tissue specimens (margin) constituting reference material in the study were used. Tissue material collected during the surgery was immediately placed in sterile tubes containing RNA *later* (Sigma) in the amount of 1 *μ*L of tissue (200 *μ*L RNA *later* per 20 mg of tissue), and, following a one-day incubation at ambient temperature, it was frozen at −80°C.

### 2.2. Microarray Analysis

The analysis of the expression profile of genes coding proteins of signal cascades activated by leptin in the samples of colorectal cancer and healthy tissue was conducted with the oligonucleotide microarray technique with application of HG_U133A plates (Affymetrix). The analysis started with comparison of the reports generated in the programme Microarray Suite Affymetrix directly after making out the fluorescence signals on the microarray. 

On the basis of the parameters characterizing the distribution of fluorescence signals on the plate, in correspondence with the recommendations of the producer of microarrays (Affymetrix “Technical Manual GeneChip Expression Analysis”), 11 transcriptomes were accepted to the comparative analysis, including 9 transcriptomes of cancer tissue (study group) and 3 transcriptomes of healthy tissue (controls). The comparative analysis of transcriptomes selected for the tested samples started with grouping of transcriptomes by data clustering (hierarchical grouping with application of Chebyshev distance and Ward's method), and the conformity of grouping was assessed on the basis of clinical, histopathological, and molecular characteristics. The transcripts which were qualified to the same group regardless of the applied criteria for classification (clinical, histopathological, or molecular) were selected to the comparative analysis. 

In the further stage of the comparative analysis, transcripts were selected from among 91 mRNA coding proteins participating in the signal transmission pathways in cells, activated through leptin binding to its receptor, differentiating tumour from healthy tissue.

Selection of 91 transcripts was based on data available in the literature and the NCBI and Affymetrix bases.

### 2.3. Statistical Analysis of the Results

The following computer programmes were used in analysis of the data obtained through the oligonucleotide microarray technique: Statistica v.6.0. Microarray Suite v.5.0, SAM (*significance analysis of microarrays*), and RMA Express which enabled data normalization consisting in taking logarithms of the data obtained in the experiment.

## 3. Results

The comparative analysis of mRNA concentration profile in the samples of normal intestine and the samples of adenocarcinoma of patients with I-IV level of clinical progression started with grouping of transcriptomes with Ward's method with application of Chebyshev distance measure ( i.e. the maximum distance in any single dimension). The obtained results indicated clear differentiation of normal transcriptomes from samples of level I and II, and III and IV of clinical progression. 

Taking into account various stages of clinical and pathological progression of colorectal cancer in the study group, three transcript groups were chosen for a comparative analysis aimed at searching for transcripts of genes differentiating colorectal cancer and reference tissues.

- Group I (4 transcriptomes) was composed of specimens of carcinoma in an early stage without distant metastases (I and II stage of clinical and pathological cancer progression according to TNM).
- Group II (3 transcriptomes) was composed of specimens assessed histopathologically as control group.
- Group III (4 transcriptomes) was composed of specimens of carcinoma in an advanced stage collected from patients with diagnosed regional lymph node metastases and with distant metastases (III and IV stage of clinical and pathological cancer progression according to TNM).

Typing of differentiating genes was performed in a panel of 91 transcripts of genes encoding proteins involved in intracellular signalling activated by leptin for samples representing stages I and II (group I) and III and IV (group III) of clinical and pathological progression of colorectal cancer as compared to the reference (group II), as well as for samples in stages III and IV of colorectal cancer progression (group III) as compared to samples representing stages I and II (group I). 

The SAM programme typed the following genes to be statistically significant in the compared groups as genes differentiating stage I and II of clinical progression of colorectal cancer: AKT1, STAT3, MCL1, and STAT5B (Table 1). 

The SAM programme typed mRNA of transcripts of the VEGFA, VEGFC, and CCND1 encoding genes to be statistically significant in the compared groups as genes differentiating stages III and IV of clinical progression of colorectal cancer (Table 2).

## 4. Discussion

The connection of cancer with the system of leptin and its receptors has to be considered as a dynamic and changeable system in relation to the external and internal environment. For instance, it is necessary to study the growth factor receptor(s) dependency. One cannot omit the presence of binding proteins, as well as the influence of other hormone and growth factors and cytokines. Cancer process is a series of molecular changes that are effects of interaction of a cell and its environment. Such processes as angiogenesis, lymphangiogenesis, cell motility, proliferation, and cell apoptosis are also important. 

Authors of numerous articles attempt to link blood leptin concentration with the risk of occurrence and the possibility of transformation of various types of cancer [11–13]. Considering the therapeutic aspect, authors ask themselves whether receptors on the surface of cancer-affected cells may be a therapeutic target for newly developed drugs, that is, monoclonal antibodies or low-molecular-weight tyrosine kinase inhibitors.

It was shown in the experimental part of the results concerning the oligonucleotide microarray analysis that as far as genes differentiating stages I–IV of clinical and pathological cancer progression from the reference are concerned, transcripts of the encoding genes AKT 1, STAT3, Mcl-1, VEGFC, and CCND1 are expressed in cancer-affected tissues, whereas the STAT5B and VEGFA protein is silenced. Bartucci et al. suggest that leptin activated of the ERK1/2 and AKT signaling pathways enhanced growth in soft agar and improved sphere formation associated with E-cadherin overexpression [14]. Hoda et al. prove that leptin may serve as a mitogenic agent and have an antiapoptotic effect on large intestine cells [15]. Similarly, Frankenberry et al. [11] and Burguera et al. [12] proved in their studies that elevated serum leptin concentrations induce breast cancer cell proliferation through activation of signal transduction pathways including MAP and PI3 kinases, leading to cancer cell growth and survival. 

Ogunwobi and Beatles [16] prove in their studies of leptin effect on colorectal cancer cells that leptin stimulates proliferation and inhibits apoptosis in human colorectal cancer cells, involving in those processes not only PI3 kinase but also JAK2 kinase and a transcription factor, the STAT3 protein. The STAT3 protein activates, as a transcription factor, antiapoptotic Mc1-1 protein expression in cells. It is postulated that elevated expression of STAT3 and its targeted genes in colorectal carcinoma may be one of malignancy markers. Studies conducted by Sharma et al. [4] and Ma et al. [17] show similarly that the endometrial cancer cell proliferation and invasion processes are induced by leptin which activates signal transduction pathways activating the STAT3 protein and the ERK2 kinase.

Moreover, leptin counteracted cytotoxic effects of 5-fluorouracil, a common colon cancer therapeutic agent [14].

In the context of the obtained results, it is necessary to extend studies of leptin and leptin receptors participation in the colorectal cancer initiation and transformation processes.

## Figures and Tables

**Table 1:** Transcripts differentiating stages I and II of colorectal cancer progression from the reference, typed from 91 transcripts by SAM. Bold: overexpressed genes; italic: genes silenced in the group representing cancer as compared to the reference.

Early (I and II) stage of colorectal cancer progression versus reference				
Genes typed by SAM				

Transcript ID	Gene symbol	Gene name	Score	*T* Test (Q)
**207163_s_at**	**AKT1**	**v-akt murine thymoma viral oncogene homolog 1**	**3.61444**	**0**
**208991_at**	**STAT3**	**signal transducer and activator of transcription 3 (acute-phase response factor)**	**3.58231**	**0**
**200798_x_at**	**MCL1**	**myeloid cell leukemia sequence 1 (BCL2-related)**	**3.52301**	**0**
*212549_at*	*STAT5B*	*signal transducer and activator of transcription 5B*	*−5.4201*	*0*

**Table 2:** Transcripts differentiating stages III and IV of colorectal cancer progression from the reference typed from 91 transcripts by SAM. Bold: overexpressed genes; italic: genes silenced in the group representing cancer as compared to the reference.

Advanced (III and IV) stage of colorectal cancer progression versus reference Genes typed by SAM				

Transcript ID	Gene symbol	Gene name	Score	*T* Test (Q)
*210512_s_at*	*VEGFA*	*Vascular endothelial growth factor A*	*−2.8837*	*0*
**209946_at**	**VEGFC**	**vascular endothelial growth factor C**	**3.1135**	**0**
**208712_at**	**CCND1**	**cyclin D1**	**2.3355**	**0**
